# A matched comparison of the patient-reported outcome measures of 38,716 total and unicompartmental knee replacements: an analysis of linked data from the National Joint Registry of England, Northern Ireland and Isle of Man and England’s National PROM collection programme

**DOI:** 10.1080/17453674.2021.1956744

**Published:** 2021-07-26

**Authors:** Hasan R Mohammad, Andrew Judge, David W Murray

**Affiliations:** a Nuffield Department of Orthopaedics, Rheumatology and Musculoskeletal Sciences, University of Oxford, Oxford, UK; b Musculoskeletal Research Unit, Bristol Medical School, University of Bristol, Level 1 Learning and Research Building, Southmead Hospital, Westbury-on-Trym, Bristol, UK

## Abstract

Background and purpose — The surgical treatment options for severe knee osteoarthritis are unicompartmental (UKR) and total knee replacement (TKR). For patients, functional outcomes are more important than revision rate. We compared the patient-reported outcome measures (PROMs) of both implant types using a large PROMs dataset.

Patients and methods — We analysed a propensity-matched comparison of 38,716 knee replacements (19,358 UKRs and 19,358 TKRs) enrolled in the National Joint Registry and the English National PROM collection programme. Subgroup analyses were performed in different age groups.

Results — 6-month postoperative Oxford Knee Score (OKS) for UKR and TKR were 38 (SD 9.4) and 36 (SD 9.4) respectively. A higher proportion of UKRs had an excellent OKS (≥ 41) compared with TKR (47% vs 36%) and a lower proportion of poor OKS (< 27) scores (13% vs. 16%). The 6-month OKS was higher in all age groups for UKR compared with TKR, with the difference increasing in older age groups. The mean 6-month EQ-5D score was 0.78 (SD 0.25) and 0.75 (SD 0.25) respectively. The improvement in EQ-5D resulting from surgery was higher for UKR than TKR both overall and in all age groups. All comparisons were statistically significant (p < 0.05).

Interpretation — UKR had a greater proportion of excellent OKS scores and lower proportion of poor scores than TKR. Additionally, the quality of life was higher for UKR compared with TKR. These factors should be balanced against the higher revision rate for UKR when choosing which procedure to perform.

The main treatments for severe knee arthritis that has failed to respond to nonoperative management are total knee replacement (TKR) and unicompartmental knee replacement (UKR). UKR offers advantages over TKR including reduced mortality and medical complications (Liddle et al. 2014), and a faster recovery, but the registries report several times higher revision rates (National Joint Registry 2018, Australian Orthopaedic Association 2019, New Zealand Joint Registry 2019). Approximately 50% of knees needing replacement are appropriate for UKR (Willis-Owen et al. 2009), yet current usage is only 10% given the higher revision rates (National Joint Registry 2018). Although there is some evidence of better functional outcomes for UKR compared with TKR, all previous studies are limited by sample size, particularly for the UKR arm (Baker et al. 2012, Liddle et al. 2015, Beard et al. 2019, Wilson et al. 2019).

In assessing risk, patients need more information than revision rate alone, which is the traditional metric for measuring joint replacement outcome (Goodfellow et al. 2010). In recent years there has been a drive towards more patient-directed outcomes. Goodman et al. (2020) found that what mattered most to patients following a knee replacement was relief of pain, restoration of function, and improved quality of life.

We compared the functional outcomes and quality of life of matched TKRs and UKRs, both overall and in different age groups, using data from 3 national datasets.

## Patients and methods

### Data sources

We performed a retrospective observational study using National Joint Registry for England Wales and Northern Ireland and Isle of Man (NJR) records linked to the Hospital Episodes Statistics Admitted Patient Care records (HES-APC) database and England’s National Patient Reported Outcome Measures (PROMs) database. The NJR was established in 2003 and is now the world’s largest arthroplasty register (National Joint Registry 2018). HES-APC records is a database of all admission episodes for patients being admitted to an NHS hospital in England (NHS Digital 2020a). From approximately 2009 onwards, NHS-funded knee replacements as part of the PROMs programme have both preoperative and 6-month postoperative PROMs recorded (NHS Digital 2020b). These include the Oxford Knee Score (OKS) (Murray et al. 2007) and quality of life index EuroQol 5 Domain index (EQ-5D) (Group 1990, Devlin et al. 2010).

The choice of time intervals by the PROMs programme was a compromise between appropriate proximity to surgery (to provide timely feedback and to avoid influence of nonoperative factors) and sufficient follow-up for comparison whilst accounting for the postoperative recovery period. Research indicates most improvement in PROMs after joint replacement occurs in the first 6 months, with only minor improvement between 6 months and 1 year (Browne et al. 2013). Long-term studies of TKR and UKR have shown that PROMs remain relatively constant after this, at least up to the 10th postoperative year (Pandit et al. 2011, Breeman et al. 2013, Williams et al. 2013).

### Data linkage

**Figure 1.**
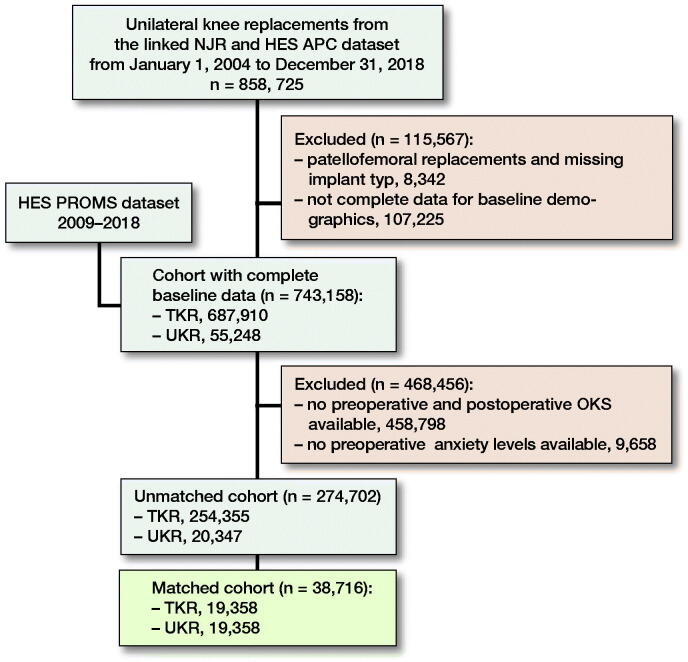
Data flowchart of dataset cleaning and merging.

Between January 1, 2004 and December 31, 2018, 687,910 TKRs and 55,248 UKRs from the NJR dataset (National Joint Registry 2018) were successfully linked to the HES APC dataset (NHS Digital 2020a) with a full set of baseline demographic and surgical factors needed for matching. Bilateral knee replacements were excluded to allow data linkage. This dataset was merged with the HES PROMs dataset, which started collecting data from approximately 2009 onwards (NHS Digital 2020b). All preoperative PROMs needed to be completed within 3 months prior to surgery or at the latest 1 month postoperatively to be regarded as robust for inclusion. Cases were excluded if either no preoperative anxiety score was available or there was not both a preoperative and postoperative OKS. The demographics of these patients excluded are summarised in Table 1 (see Supplementary data). There were 254,355 TKRs and 20,347 UKRs meeting the above criteria, making them eligible for inclusion (Figure 1). Datasets were linked using pseudo-anonymised identification numbers.

**Table 1.** Patients without PROMs data (n = 468,456) after linking NJR data to HES PROMs dataset. Values are count (%) unless otherwise specified

	TKR	UKR
Covariate	(n = 433,555)	(n = 34,901)
Admission type		
Elective	432,181 (100)	34,842 (100)
Emergency	982 (0)	35 (0)
Other	392 (0)	24 (0)
Sex		
Female	252,531 (58)	16,717 (48)
Male	181,024 (42)	18,184 (52)
Age at surgery,		
mean (SD)	70.2 (9.5)	63.7 (10.0)
BMI, n	257,360 (	22,024 (
mean (SD)	31.1 (5.6)	30.5 (5.2)
Primary diagnosis		
Primary OA	414,553 (96)	34,299 (98)
Primary OA and other	5,387 (1)	236 (1)
Other	13,615 (3)	366 (1)
Charlson comorbidity index		
None	302,652 (70)	26,106 (75)
Mild	92,549 (21)	6,724 (19)
Moderate	25,839 (6)	1,495 (4)
Severe	12,515 (3)	576 (2)
ASA grade		
1	40,922 (9)	6,680 (19)
2	306,864 (71)	24,579 (70)
3 or above	85,769 (20)	3,642 (11)
Ethnicity		
White	403,614 (93)	33,296 (95)
Black (Caribbean)	3,495 (1)	150 (1)
Black (African)	2,086 (1)	113 (0)
Black (other)	838 (0)	33 (0)
Indian	12,124 (3)	554 (2)
Pakistani	4,852 (1)	211 (1)
Bangladeshi	379 (0)	20 (0)
Chinese	341 (0)	17 (0)
Other	5,826 (1)	507 (1)
Rural/urban classification		
Urban	332,812 (77)	24,934 (72)
Town/fringe	48,461 (11)	4,280 (12)
Village/hamlet	52,282 (12)	5,687 (16)
Indices of multiple deprivation		
Most deprived (20%)	73,212 (17)	4,328 (12)
More deprived (20–40%)	84,133 (19)	6,155 (18)
Middle-point	95,016 (22)	7,728 (22)
Less deprived (20–40%)	95,598 (22)	8,130 (23)
Least deprived (20%)	85,596 (20)	8,560 (25)

### Propensity matching

There were substantial differences in baseline characteristics between TKR and UKR groups (Table 2). Logistic regression was used to generate a propensity score representing the probability that a patient received a UKR and were generated from patient demographics and surgical factors. All patient and surgical factors in Table 2 were used for matching, apart from BMI, which had a large proportion of missing data. This is a well-recognised approach (Matharu et al. 2017, Matharu et al. 2018, Mohammad et al. 2020a, 2020b). Surgical factors included surgeon caseload, defined as the average number of primary knee replacements performed per year as described previously (Liddle et al. 2016, Mohammad 2020a). The algorithm used matched 1:1 on the logit of the propensity score with a 0.02-SD calliper width. Greedy matching without replacement was used given its superior performance for estimating treatment effects (Austin 2009a). Standardized mean differences (SMDs) were examined both before and after matching to assess for any imbalance between groups, with SMDs of > 10% suggestive of covariate imbalance (Austin 2009b). After matching, 38,716 knee replacements (19,358 TKRs and 19,358 UKRs) were included for analysis.

**Table 2.** Baseline characteristics before matching TKRs and UKRs. Values are count (%) unless otherwise specified

	Unmatched cohort			Matched cohort		
	TKR	UKR		TKR	UKR	
Covariate	(n = 254,355)	(n = 20,347)	SMD	(n = 19,358)	(n = 19,358)	SMD
Admission type						
Elective	254,178 (100)	20,337 (100)	0.01	19,349 (100)	19,349 (100)	0.01
Emergency	163 (0)	10 (0)		8 (0)	9 (0)	
Other	14 (0)	0 (0)		1 (0)	0 (0)	
Sex						
Female	145,049 (57)	9,611 (47)	0.20	9,083 (47)	9,206 (48)	0.01
Male	109,306 (43)	10,736 (53)		10,275 (53)	10,152 (52)	
Age at surgery,						
mean (SD)	70.2 (8.8)	64.7(9.3)	0.60	64.9 (9.0)	65.1 (9.3)	0.02
BMI, n	192,787 (	15,919 (		14,815 (	15,059 (	
mean (SD)	30.9 (5.4)	30.2 (4.9)	0.14	31.0 (5.3)	30.2 (4.9)	0.16
Primary diagnosis						
Primary OA	246,026 (97)	19,993 (98)	0.10	19,011 (98)	19,013 (98)	0.01
Primary OA and other	2,645 (1)	129 (1)		139 (1)	125 (1)	
Other	5,684 (2)	225 (1)		208 (1)	220 (1)	
Charlson comorbidity index						
None	177,003 (70)	14,997 (74)	0.10	14,343 (74)	14,245 (74)	0.02
Mild	54,018 (21)	3,963 (19)		3,778 (20)	3,787 (20)	
Moderate	16,160 (6)	972 (5)		857 (4)	921 (4)	
Severe	7,174 (3)	415 (2)		380 (2)	405 (2)	
Ethnicity						
White	243,425 (96)	19,759 (97)	0.09	18,781 (97)	18,788 (97)	0.01
Black (Caribbean)	1,291 (1)	70 (0)		70 (0)	68 (0)	
Black (African)	849 (0)	39 (0)		41 (0)	39 (0)	
Black (other)	379 (0)	19 (0)		17 (0)	18 (0)	
Indian	4,499 (2)	217 (1)		228 (1)	213 (1)	
Pakistani	1,280 (0)	51 (0)		45 (0)	50 (0)	
Bangladeshi	113 (0)	4 (0)		6 (0)	4 (0)	
Chinese	175 (0)	5 (0)		5 (0)	5 (0)	
Other	2,344 (1)	183 (1)		165 (1)	173 (1)	
Rural/urban classification						
Urban	187,601 (74)	14,044 (69)	0.12	13,520 (70)	13,451 (70)	0.008
Town/fringe	31,579 (12)	2,612 (13)		2,458 (13)	2,476 (13)	
Village/hamlet	35,175 (14)	3,691 (18)		3,380 (17)	3,431 (17)	
Indices of multiple deprivation						
Most deprived (20%)	34,627 (14)	1,961 (10)	0.18	1,906 (10)	1,917 (10)	0.006
More deprived (20–40%)	45,285 (18)	3,111 (15)		3,010 (16)	2,996 (16)	
Middle-point	56,721 (22)	4,540 (22)		4,295 (22)	4,324 (22)	
Less deprived (20–40%)	60,782 (24)	4,957 (24)		4,779 (24)	4,737 (24)	
Least deprived (20%)	56,940 (22)	5,778 (29)		5,368 (28)	5,384 (28)	
Surgeon caseload of primary knee surgery practice						
Cases/years, mean (SD)	80.5 (48.2)	97.6 (50.6)	0.35	96.7 (55.1)	96.7 (50.5)	0.001
Primary complexity						
Normal	254,328 (100)	20,344 (100)	0.004	19,354 (100)	19,355 (100)	0.004
Complex	27 (0)	3 (0)		4 (0)	3 (0)	
ASA grade						
1	22,257 (9)	3,646 (18)	0.34	3,418 (17)	3,294 (17)	0.02
2	190,181 (75)	14,901 (73)		14,274 (74)	14,297 (74)	
3 or above	41,917 (16)	1,800 (9)		1,666 (9)	1,767 (9)	
VTE prophylaxis—chemical						
LMWH (± other)	179,562 (71)	13,586 (67)	0.10	12,708 (66)	12,882 (67)	0.02
Aspirin only	12,338 (5)	1,370 (7)		1,316 (7)	1,283 (7)	
Other	55,739 (22)	4,895 (24)		4,855 (25)	4,704 (24)	
None	6,716 (2)	496 (2)		479 (2)	489 (2)	
VTE prophylaxis—mechanical						
Any	242,433 (95)	19,775 (97)	0.10	18,781 (97)	18,793 (97)	0.004
None	11,922 (5)	572 (3)		577 (3)	565 (3)	
Fixation						
Cemented	246,269 (97)	14,932 (74)	0.70	15,023 (78)	14,926 (77)	0.01
Cementless	7,209 (3)	4,950 (24)		3,920 (20)	4,014 (21)	
Hybrid	877 (0)	465 (2)		415 (2)	418 (2)	

VTE = venous thromboemolism

### Outcomes of interest

Outcomes of interest were: (1) preoperative OKS and EQ-5D scores, (2) 6-month postoperative OKS and EQ-5D scores, and (3) difference in OKS and EQ-5D scores postoperatively and preoperatively. Subgroup analyses were performed in 4 different age groups as per the NJR (National Joint Registry 2018); < 55 years, 55–64 years, 65–74 years, and ≥ 75 years.

The OKS has 12 items relating to knee pain and function, presented as an overall score between 0 and 48 (Murray et al. 2007). Mean OKS scores are reported with the proportion attaining excellent (≥ 41), good (34–41), fair (27–33), and poor (< 27) results defined by Kalairajah et al. (2005). Various estimates of the minimal clinically important difference (MCID) for the OKS have been made; this is considered to be between 3 and 5 points (Beard et al. 2015). The EQ-5D comprises 5 questions concerning mobility, selfcare, activities of daily living, pain, and anxiety/depression. These answers can be presented as a weighted overall index from 1 (perfect health) to –0.594 (worst possible state) (Group 1990, Devlin 2010).

### Statistics

Given that PROMs scores were not normally distributed, appropriate nonparametric tests were used. To compare pre- and postoperative scores within TKR and UKR groups we used the Wilcoxon signed rank test. To compare TKR and UKR scores the Mann–Whitney test was performed. Locally weighted scatterplot smoothing (LOWESS) curves (Cleveland 1979) were plotted to explore the relationship between preoperative and postoperative PROMs. For clarity purposes the scatter points are suppressed in the plots presented. The percentage of the possible change (PoPC) was calculated as described previously (Kiran et al. 2014). This expresses the actual change attained as a percentage of the possible change, for example for a preoperative OKS of 20 with postoperative score of 40. The actual change is 20 points and the possible change is 48–20 = 28. Therefore the PoPC is 20/28*100 = 71.4%.

All statistical analyses were performed using Stata (Version 15.1; StataCorp, College Station, TX, USA) except propensity score matching, which was performed using R (Version 3.4.0; R Foundation for Statistical Computing, Vienna, Austria). P-values of < 0.05 were considered significant.

### Ethics, funding, and potential conflicts of interest

This study was approved by the NJR Research subcommittee and had ethical approval from the South Central Oxford B Research Ethics Committee (19/SC/0292). The linkage of the datasets was approved by the Confidentiality Advisory Group (19/CAG/0054). Financial support has been received from Zimmer Biomet.

HRM was supported by the Henni Mester Scholarship at University College, Oxford University and the Royal College of Surgeons’ Research Fellowship. AJ was supported by the NIHR Biomedical Research Centre at the University Hospitals Bristol NHS Foundation Trust and the University of Bristol.

## Results

**Figure 2.**
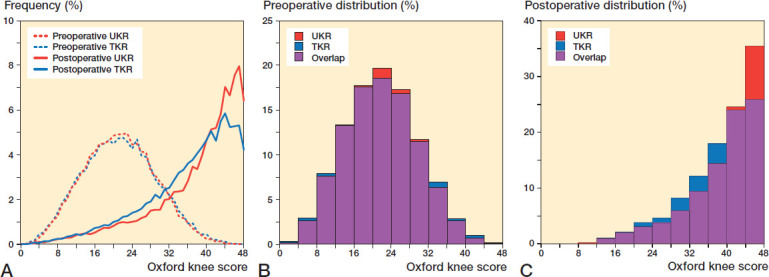
Distribution of OKS in TKR and UKR groups: (A) preoperative and postoperative line graph; (B) preoperative histogram; (C) postoperative histogram.

The unmatched cohort consisted of 254,355 TKRs and 20,347 UKRs with several statistically significant baseline differences between groups (Table 2). The matched study group consisted of 38,716 knee replacements (19,358 TKRs, 19,358 UKRs), which were well balanced (Table 2). The distribution of the OKS scores is illustrated in Figure 2.

**Figure 3.**
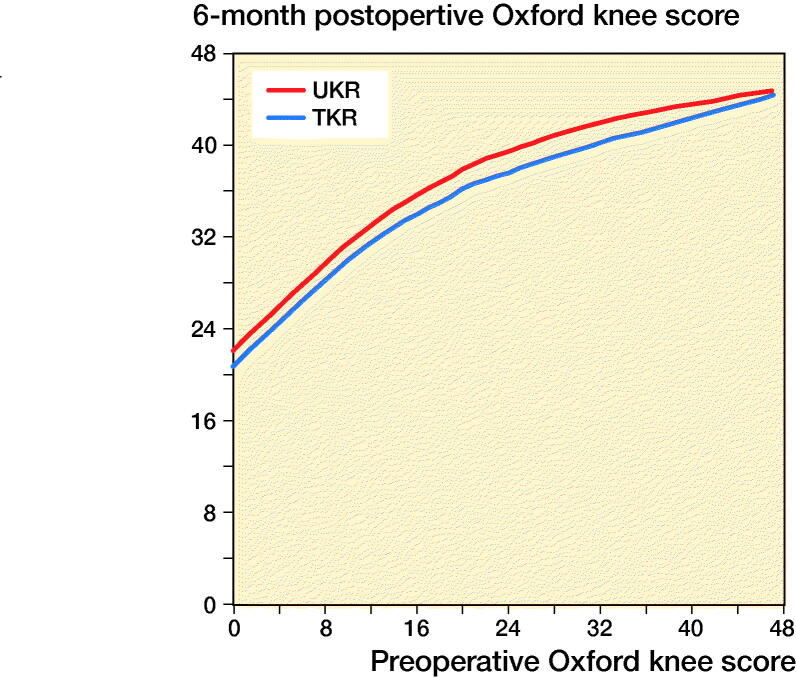
LOWESS curve showing the relationship between preoperative and postoperative Oxford Knee Score for TKR and UKR groups.

**Figure 4.**
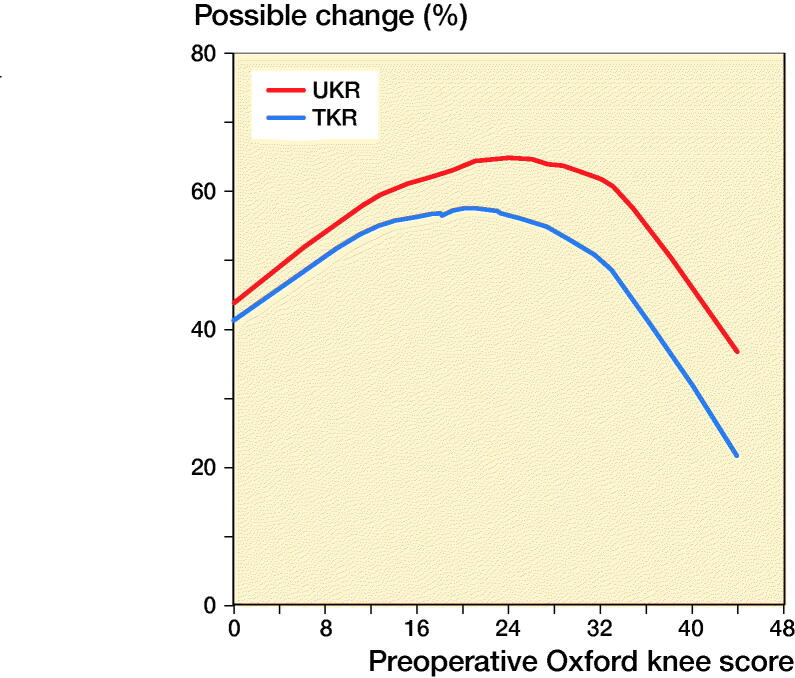
LOWESS curve of percentage of the possible change for TKR and UKR in relation to preoperative OKS.

LOWESS curves showed as preoperative OKS increased as did the postoperative score, with similar gradients for both implants. A ceiling effect was visible for the higher preoperative scores (Figure 3). For any given preoperative score, the postoperative score was higher for UKR than TKR. Figure 4 shows how the PoPC was influenced by preoperative score through LOWESS curves. For all preoperative scores the PoPC was greater for UKR than TKR. The higher the preoperative score the larger the differences.

**Figure 5.**
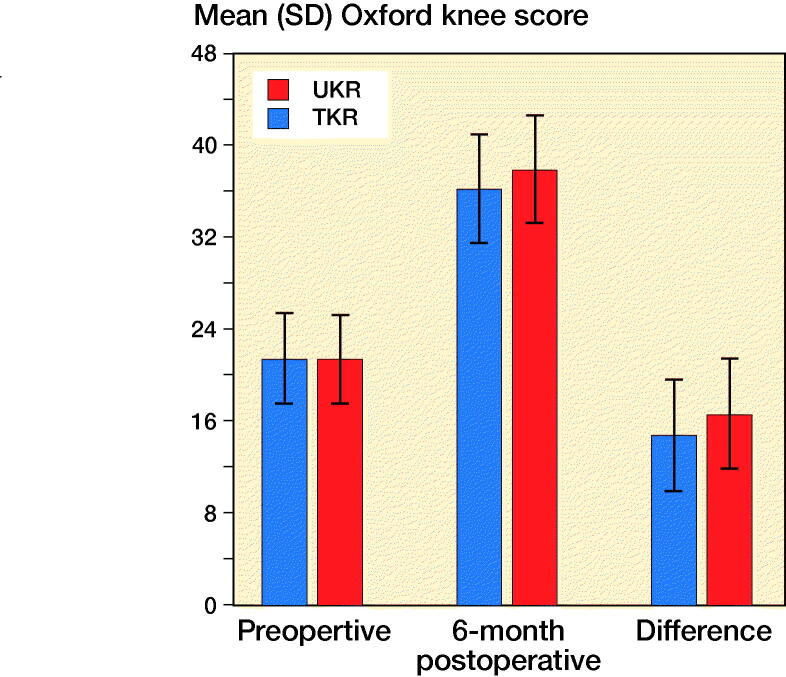
Comparison of mean OKS in matched cohort of TKR and UKRs. Error bars represent SD.

The mean preoperative OKS for the TKR and UKR groups was similar, at 21 (SD 7.9) and 21 (SD 7.7). Both groups showed statistically significant improvements in their 6-month postoperative scores (p < 0.001) to 36 (SD 9.4) and 38 (SD 9.4) respectively. The UKR group had a statistically significantly higher (p < 0.001) 6-month postoperative score by 1.7 points. The TKR group gained 15 points (SD 9.8) postoperatively whereas the UKR group gained 17 points (SD 9.6), with the difference being statistically significant (p < 0.001) (Figure 5).

The UKR groups had a statistically significantly higher proportion of excellent OKS compared with TKR (47% vs. 36%, p < 0.001) 6 months postoperatively (Table 3). The UKR group had a statistically significantly lower proportion of poor scores than TKR at 6 months postoperatively (13% vs. 16%, p < 0.001) (Table 3).

**Table 3.** Proportion of OKS (Kalairajah et al. classification)

Factor	TKR (n = 19,358)	UKR (n = 19,358)	
	n (%)	n (%)	p-value
Preoperative OKS categorisation			
Poor	14,244 (74)	14,391 (74)	0.5
Fair	3,780 (20)	3,820 (20)	0.7
Good	1,223 (6)	1,070 (6)	0.002
Excellent	111 (0)	77 (0)	0.01
Postop OKS categorisation:			
Poor	3,115 (16)	2,538 (13)	< 0.001
Fair	3,071 (16)	2,356 (12)	< 0.001
Good	6,200 (32)	5,442 (28)	< 0.001
Excellent	6,972 (36)	9,022 (47)	< 0.001

Comparisons between implant types were performed using the chi-square proportional test.

**Figure 6.**
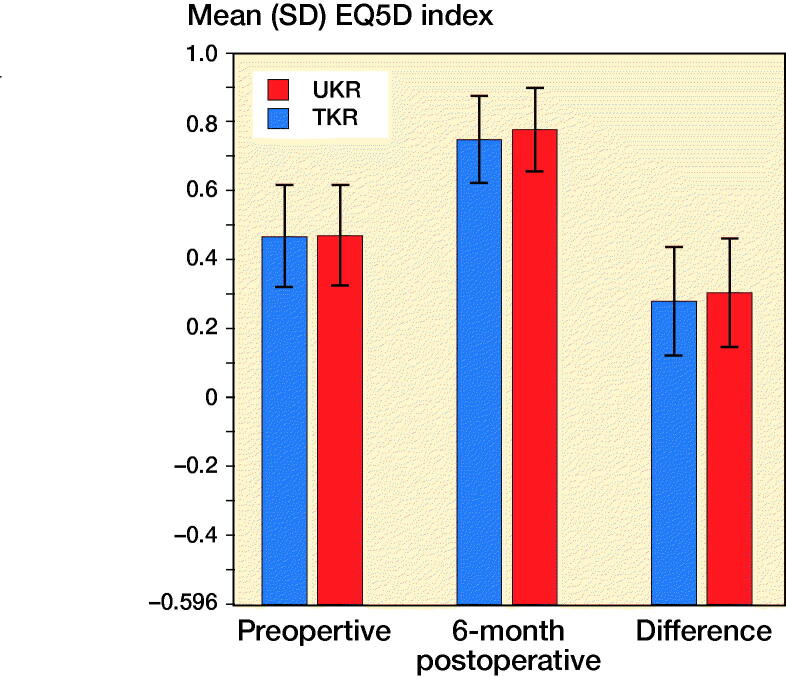
Comparison of mean EQ-5D index in matched cohort of TKR and UKRs. Error bars represent SD.

The mean preoperative EQ-5D index for the TKR and UKR groups was 0.47 (SD 0.30) and 0.47 (SD 0.30) respectively with this difference being non-statistically significant (p = 0.3). Both groups showed a statistically significant improvement in their 6-month scores (p < 0.001) to 0.75 (SD 0.25) and 0.78 (SD 0.25) respectively. The TKR group gained 0.28 (SD 0.32) points postoperatively whereas the UKR group gained 0.31 (SD 0.31) with the difference being statistically significant (p < 0.001) (Figure 6).

### Effect of age on PROMS in the matched cohort

Age groups were stratified into 4 groups as per the NJR: (1) < 55 years (2,569 TKRs, 2,763 UKRs); (2) 55–64 years (6,581 TKRs, 6,256 UKRs); (3) 65–74 years (7,415 TKRs, 7,182 UKRs); and (4) ≥ 75 years (n = 2,793 TKRs, 3,157 UKRs).

Preoperative OKS was similar between TKR and UKR across all age groups. Younger age groups had poorer OKS than older groups, reflecting the higher threshold to operate in younger patients (Table 4, see Supplementary data). For both TKR and UKR all age groups showed statistically significant improvements postoperatively compared with preoperatively (p < 0.001) (Table 4, see Supplementary data). UKR gained more points postoperatively compared with TKR across all age groups (p < 0.001), with this difference increasing with age (Table 4, see Supplementary data). The 6-month OKS was higher in all age groups for UKR compared with TKR (p < 0.001) with the difference increasing in the older age groups (Table 4, see Supplementary data).

**Table 4.** Pre- and postoperative OKS in different age groups for TKR and UKR groups

Age group	TKR OKS			UKR OKS			6-months difference
	preoperative	at 6 month	diff.	preoperative	at 6 month	diff.	
< 55	18.6 (7.8)	33.5 (10.9)	14.9	19.0 (7.6)	34.8 (10.9)	15.8	1.3
55–64	20.6 (7.7)	35.5 (9.7)	14.9	20.7 (7.5)	37.2 (9.8)	16.5	1.7
65–74	22.6 (7.8)	37.5 (8.6)	14.9	22.4 (7.5)	39.4 (8.3)	17.0	1.9
≥ 75	22.6 (8.1)	36.9 (8.7)	14.3	22.3 (7.8)	38.7 (8.4)	16.4	1.8

Preoperatively the proportions of poor, fair, good, and excellent OKS were similar between TKR and UKR for all age groups (Table 5, see Supplementary data). At 6 months postoperatively there was a greater proportion of excellent and a lower proportion of poor scores for UKR compared with TKR across all age groups (Table 5, see Supplementary data). The proportion of excellent scores was 6%, 12%, 12%, and 10% higher in UKR compared with TKR for the < 55 years, 55–64 years, 65–74 years, and ≥ 75 years groups respectively. The proportion of poor OKS scores was 3–4% lower in UKR compared with TKR across all age groups.

**Table 5.** Proportion of poor (< 27), fair (27–33), good (34–41), and excellent (≥ 41) OKS (Kalairajah et al. classification) in different age groups for TKR and UKR groups. Values are count (%)

		TKR (n = 19,358)				UKR (n = 19,358)		
Age group	Poor	Fair	Good	Excellent	Poor	Fair	Good	Excellent
Preoperative								
< 55	2,166 (84)	304 (12)	89 (4)	10 (0)	2,285 (83)	385 (14)	84 (3)	9 (0)
55–64	5,116 (78)	1,128 (17)	302 (5)	35 (0)	4,879 (78)	1,058 (17)	301 (5)	18 (0)
65–74	5,079 (69)	1,692 (23)	595 (8)	49 (1)	5,015 (70)	1,667 (23)	466 (6)	34 (1)
≥ 75	1,883 (67)	656 (24)	237 (8)	17 (1)	2,212 (70)	710 (22)	219 (7)	16 (1)
Postoperative at 6 months								
< 55	648 (25)	434 (17)	751 (29)	736 (29)	614 (22)	405 (15)	772 (28)	972 (35)
55–64	1,216 (19)	1,069 (16)	2,104 (32)	2,192 (33)	957 (15)	767 (12)	1,744 (28)	2,788 (45)
65–74	882 (12)	1,119 (15)	2,394 (32)	3,020 (41)	643 (9)	815 (11)	1,960 (27)	3,764 (53)
≥ 75	369 (13)	449 (16)	951 (34)	1,024 (37)	324 (10)	369 (12)	966 (31)	1,498 (47)

Preoperatively the EQ-5D score was similar between TKR and UKR across age groups (Table 6, see Supplementary data). For both TKR and UKR younger age groups had poorer EQ-5D scores than older groups. For both TKR and UKR all age groups showed statistically significant improvements postoperatively compared with preoperatively (p < 0.001) although UKR gained more points postoperatively compared with TKR across all age groups (p < 0.001) except the < 55 group (p = 0.5) (Table 6, see Supplementary data). The 6-month EQ-5D was higher in all age groups for UKR compared with TKR (p < 0.001) (Table 6, see Supplementary data).

**Table 6.** Pre- and postoperative EQ5D index scores in different age groups for TKR and UKR groups

Age group	TKR OKS			UKR OKS			6-months difference
	preoperative	at 6 month	diff.	preoperative	at 6 month	diff.	
< 55	0.375 (0.322)	0.678 (0.297)	0.303	0.397 (0.318)	0.701 (0.298)	0.304	0.023
55–64	0.450 (0.303)	0.727 (0.260)	0.277	0.454 (0.301)	0.756 (0.259)	0.302	0.029
65–74	0.506 (0.283)	0.789 (0.218)	0.283	0.506 (0.279)	0.817 (0.216)	0.311	0.028
≥ 75	0.513 (0.276)	0.783 (0.216)	0.270	0.504 (0.281)	0.808 (0.211)	0.304	0.025

## Discussion

This is the largest study comparing the PROMs of TKR and UKR and helps provide answers to outcome metrics patients find most important (Goodman et al. 2020). After matching, a substantially higher proportion of UKRs had an excellent OKS compared with TKR (47% vs. 36%) and a lower proportion of poor scores (13% vs. 16%). This is important, given that currently 1 in 5 patients who undergo a total knee replacement are dissatisfied with their knee replacement (Beswick et al. 2012, Price et al. 2018). This number would likely be lower if more patients suitable for UKR had UKR surgery, which by some estimates could be up to 50% (Willis-Owen et al. 2009).

The 6-month postoperative OKS of UKR was higher than TKR by 2 points. This difference is similar to the TOPKAT (Beard et al. 2019) randomised control trial, suggesting that it is a real difference. Although the magnitude of the difference is below the suggested MCID for the OKS, given the skewed nature of the outcome scores (Figure 2), together with the ceiling effect of the OKS (Dawson et al. 2014), this does not mean that the difference is unimportant. Indeed, its importance is highlighted by finding that the relative risk (1.3) of having an excellent score is 30% higher following a UKR rather than a TKR and the relative risk (0.81) of having a poor score is 20% less following UKR. Additionally, for any given preoperative OKS, a greater PoPC postoperatively was observed for UKR than for TKR (Figure 4). This was particularly marked in patients with higher preoperative OKS.

In all age groups except the < 55 years group, the average 6-month postoperative OKS was about 2 points greater with UKR than TKR and UKR were about 30% more likely to have an excellent OKS and 20% less likely to have a poor OKS. In the < 55 age group the difference in 6-month postoperative OKS between TKR and UKR groups was only 1.3 points. Also, in this age group UKR were 20% more likely to have an excellent outcome and 12% less likely to have a poor outcome. It is not clear why this is. It may be because the preoperative scores were much lower in this group, and, as can be seen in the graph comparing pre- and postoperative OKS (Figure 3), with lower preoperative scores the difference between UKR and TKR is smaller. It may also be that in this age group more UKR patients had early stages of osteoarthritis (without bone-on-bone arthritis) and these patients tend not to do well (Kennedy et al. 2020a). Despite this, in all age groups UKR have better function than TKR, which justifies using UKR in all age groups. This is particularly important in the elderly as only 4% of knee replacements in patients older than 75 years are UKR (National Joint Registry 2018), when approximately one-third of knee replacements are appropriate for UKR (Kennedy et al. 2020b). Therefore approximately 10 times as many UKRs could be done in this age group.

This study has also shown UKR offers better 6-month quality of life with EQ-5D scores both overall and on age subgroup analyses. Overall and in all age groups the EQ-5D index was between 0.02 and 0.03 points higher for UKR compared with TKR. This is close to the lower limit of the predicted MCID for the EQ-5D index, which is considered to range between 0.03 and 0.54 points (Coretti et al. 2014). When determining quality of life improvement the index is summated annually, so, over the time period the devices are implanted, the differences are likely to be appreciable and well above the MCID.

Our results agree with those reported from Liddle et al. (2015) who found that UKR had higher 6-month OKS and EQ-5Ds than matched TKRs. In contrast they are different from those of Baker et al. (2012) who found no difference in the PROMs gained by UKR and TKR in an analysis adjusted for case-mix and preoperative score. However, there were only 505 UKRs in the Baker study, suggesting that this study was underpowered compared with the Liddle study (3,519 UKRs), which had about 7 times as many UKR, and our study, which had about 40 times as many UKR (n = 19,358).

This is the largest study comparing the PROMs of matched UKR and TKR and the first to perform analyses in different age groups. The main study strengths are that we used an unselected registry sample, which reduces the chances of selection bias. By linking datasets various confounding factors were matched, allowing fair comparison. The main limitation is that this is a retrospective study with 6-month postoperative scores with some evidence existing (Browne et al. 2013) of slight further increases (below the MCID) in OKS between 6 and 12 months postoperatively. However, most improvement in PROMs after joint replacement occurs in the first 6 months (Pandit et al. 2011, Breeman et al. 2013, Browne et al. 2013, et al.Williams 2013). Additionally, matching can reduce the generalisability of findings, but given we were able to match virtually all the UKR to TKR this is unlikely to be an issue. Finally, we were only able to match using variables collected in the databases. There could be unaccounted variables that could lead to some residual confounding.

## Conclusion

Surgeons have traditionally made the decision on which implant to use based on relative revision rates; however, patients are much more concerned about functionality (Goodman et al. 2020). This study shows that UKR offers superior functional outcomes and quality of life across all age groups. Although the absolute mean difference in OKS is below the MCID, the likelihood of an excellent OKS is about 30% higher for UKR and the likelihood of a poor OKS is about 20% lower for UKR. We recommend that the findings of this study are discussed with patients alongside the increased risk of UKR revision to help patients make more informed decisions about their care. 

## Supplementary data

Tables 1 and 4–6 are available as supplementary data in the online version of this article, http://dx.doi.org/10.1080/17453674.2021.1956744
